# 3D-Printing Techniques on the Development of Multiparameter Sensors Using One FBG

**DOI:** 10.3390/s19163514

**Published:** 2019-08-11

**Authors:** Arnaldo G. Leal-Junior, Camilo Díaz, Carlos Marques, Anselmo Frizera, Maria José Pontes

**Affiliations:** 1 Mechanical Engineering Department, Federal University of Espírito Santo, Fernando Ferrari avenue, 29075-910, Vitória-ES, Brazil; 2 Graduate Program on Electrical Engineering, Federal University of Espírito Santo, Fernando Ferrari avenue, 29075-910, Vitória-ES, Brazil; 3 Instituto de Telecomunicações and Physics Department & I3N, Universidade de Aveiro, Campus Universitário de Santiago, 3810-193 Aveiro, Portugal

**Keywords:** optical fiber sensors, fiber bragg gratings, additive layer manufacturing, multiparameter sensing

## Abstract

We report the development of a fiber Bragg grating (FBG) sensor for multiparameter sensing using only one FBG. The FBG was half-embedded in a 3D-printed structure, which resulted in a division of the grating spectrum creating two peaks with different sensitivities with respect to different physical parameters. A numerical analysis of the proposed technique was performed using the coupled-mode theory with modified transfer matrix formulation. Then, experimental analyses were performed as function of temperature, strain and force, where the peaks showed different sensitivities in all analyzed cases. Such results enable the application of a technique for simultaneous measurement of multiple physical parameters using both peaks and the full width half maximum of the FBG embedded in a 3D structure. In the simultaneous multiparameter assessment, the proposed sensor system was able to estimate the three tested parameters (strain, temperature and force) with relative errors as low as 4%.

## 1. Introduction

Since their first report in 1978 [1], fiber Bragg gratings (FBGs) have experienced a continuous growth on applications such as fiber lasers, dispersion compensation, band-rejection filters and wavelength selective devices as summarized in [1]. In the last decades, the tunability properties of the Bragg wavelength as a function of the temperature and strain begun to be explored in sensors applications [2]. Advantages like compactness, lightweight, chemical stability, electromagnetic immunity, multiplexing capabilities and wavelength-encoded data have led to the rapid widespread of FBG sensors in different application fields, e.g., industrial processes [3], structural health monitoring [4], biomedical [5] and robotics [6].

In such applications, FBGs can be embedded in different materials and structures to measure not only strain and temperature, but also liquid level [7], humidity [8] and force [6]. In addition, as FBGs with different Bragg wavelengths can be inscribed in the same fiber only by changing the grating period. Thus, multiparameter and multipoint measurements can be performed using only one fiber in a quasi-distributed sensing configuration with spatial resolution of a few centimeters.

Concurrently with the widespread of FBG sensors, advancements in additive layer manufacturing (ALM) techniques also evolved, especially with the popularization of 3D-printing. This new manufacturing technique has advantages such as low relative cost, flexibility on the manufacturing process, possibility of recycling the wasted material, high customization and with no need for additional post-fabrication [9]. Owing to these advantages, 3D-printing has been used as a tool on the development of FBG-based sensors. To that extent, FBGs are embedded in 3D-printed structures for the assessment of structural deformation [10], plantar pressure platforms [11] and human-robot interaction forces assessment [6]. The force and temperature responses of FBGs have been explored under different infill densities [12] and using different 3D-printing materials [13], where the spectral characteristics of embedded FBGs were also explored [14].

As the accuracy requirements in control systems for many practical applications increase, the performance demands and the number of sensors for the instrumentation of industrial plants also substantially increase [15]. Although the FBGs can be multiplexed, the number of sensors is limited to some constraints such as spectral range of the light source and spectrometers, spectral width of the FBG spectra, sensor sensitivities and resolution. In order to optimize the spectrum’s bandwidth, which enables using more sensors in quasi-distributed configurations, different approaches for simultaneous measurements of different parameters have been proposed [16]. Such approaches resulted also in the increase of the autonomy and compactness of the sensors systems [17]. In this way, techniques such as hybrid sensors [7], coated superstructure fiber grating [18], grating assembly in different structures [16], material transient analysis [17] and evaluation of the grating spectra at different loading conditions [19] have been used for multiparameter measurements using only one FBG. These approaches have also been explored on the temperature compensation [20,21] as well as in the study of epoxy resins [22]. However, these techniques have either complex manufacturing, sensor assembly or signal processing. As another examples of optical fiber-based multiparameter sensors, hybrid setups with FBGs and long period gratings have been proposed [23], hybrid cavity interferometers [24] as well as FBGs with Fabry-Perot interferometers [25]. 

Considering this background, this paper proposes the application ALM techniques on the development of multiparameter sensors using FBGs. The FBGs are half-embedded in a 3D-printed structure, which results in a division of the grating spectra, where the two resultant peaks and the full width half maximum (FWHM) have different sensitivities to physical parameters. The technique is numerically evaluated using the coupled-mode theory with modified transfer matrix formulation [26]. Then, the manufactured sensors are experimentally verified with respect to temperature, strain and transverse force variations and a technique for simultaneously measure all three parameters using a coefficient matrix was validated. The maturity of the FBG inscription in conjunction with the employed customizable prototyping methods enable the fast development of highly customizable sensor systems. Thus, one can envisage this technique being used in applications where a multitude of sensors is required or when a multiple parameters have to be measured in micrometer resolution.

## 2. Sensors Preparation and Operation Principle 

**Figure 1:**
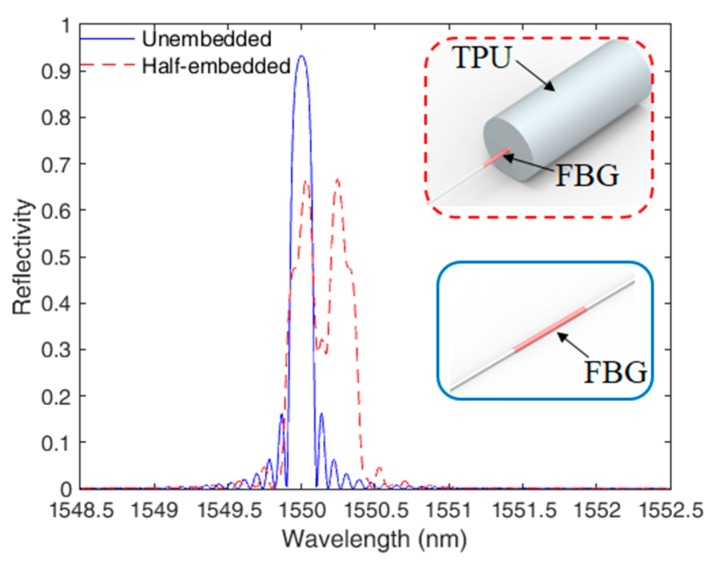
FBG spectra for unembedded and half-embedded cases. Inset shows a schematic representation of the embedment on the 3D-printed structure.

The FBGs were inscribed at Instituto de Telecomunicações (IT-Portugal) in a photosensitive single mode silica fiber GF1B (Thorlabs, NJ, USA). The phase mask technique was used on the gratings inscription through a KrF Bragg Star Industrial-LN excimer laser operating at 248 nm with a pulse duration of 15 ns. Regarding the parameters used in the grating inscription, the pulse energy is about 5 mJ, whereas the repetition frequency is 500 Hz, which resulted in an FBG at the 1550 nm wavelength region with 10 mm of physical length. After the FBG inscription, a cylindrical structure 3D-printed using the 3D printer Sethi3D S3 (Sethi, Brazil). The thermoplastic polyurethane (TPU) was the material used on the printing with an infill density of 70%, where such material was used due to its higher flexibility. When the 3D printing reaches its half layer, the fiber is positioned in such a way that only half of the grating is embedded on the 3D-printed TPU structure (see Figure 1). It is worth noting that if other material is used, such as polylatic acid (PLA) or Acrylonitrile butadiene styrene (ABS), the sensor responses will be different, since it depends on the material properties as evaluated in [13]. In this case, we used the TPU due to its lower temperature response time (1.3 s) when compared with the PLA samples (1.6 s) [13]. In addition, the TPU has lower Young’s modulus than PLA and its flexibility enables the use in transverse force sensing without a high reduction of its sensitivity. It is worth noting that using materials with Young’s modulus in the order of GPa (such as ABS and PLA), there is a sensitivity reduction on transverse force sensing when FBG embedded in ABS was compared with unembedded FBGs as demonstrated in [12]. Moreover, the shape and dimensions of the 3D printed structure can also influence the sensors responses. For this reason, 3D printed structures with the same shape and dimensions were used in all tests. The structure has a cylindrical shape with 10 mm diameter and a length of 25 mm, which was chosen in order to increase the sensitivity difference between the embedded and unembedded regions of the FBG, since the sensitivity variation can be related to the 3D-printed dimensions and material infill [12].

For the FBG embedment on the 3D-printed structure, there will be a strain applied on the grating due to the strain distribution on the embedded region. The embedment process leads to a redshift and a slight reduction on the grating reflectivity as demonstrated in [12]. Thus, if only half of the FBG is embedded on the 3D-printed structure, the strain on the FBG will be limited to half of the grating. Hence, one can expect that there will be a division on the grating spectrum, resulting in two peaks, one related to the embedded region, and the other to the unembedded section of the grating.

This assumption is firstly evaluated through a numerical analysis using the modified coupled-mode theory with modified transfer matrix formulation, as thoroughly described in [26]. In this case, we consider only the mode for forward (*R(z)*) and backward propagation (*S(z)*), where their amplitudes in the direction along the grating are presented as follows:

(1) $$\frac{dR(z)}{dz}=i\sigma R(z)+ikS(z)$$

(2) $$\frac{dS(z)}{dz}=-i\sigma S(z)-ikR(z)$$

where *k* and *σ* are “ac” and “dc” coupling coefficients, described in Equations (3) and (4), respectively.

Considering Equations (3) and (4), neff is the effective refractive index, λ is the wavelength, λB is the Bragg wavelength and periodical perturbation in the FBG effective refractive index (δneff) is defined for uniform FBGs as follows:

(3) $$k=\frac{\pi }{\lambda }\nu \bar{\delta }n_{eff}$$

(4) $$\sigma =2\pi n_{eff}\left(\frac{1}{\lambda }-\frac{1}{\lambda_{B}}\right)+\frac{2\pi }{\lambda }\bar{\delta }n_{eff}-\frac{1}{2}\Phi^{\prime }(z)$$

(5) $$\delta n_{eff}=\bar{\delta }n_{eff}\left[1+\nu \cos\left(\frac{2\pi }{\Lambda_{0}}z\right)\right]$$

where $\bar{\delta }n_{eff}$ is the average index variation over a grating period, Λ*_0_* is the nominal period, *ν* is the fringe visibility and *z* is the length along the grating. As it is well-known, the Bragg wavelength varies with temperature (Δ*T*) and strain -distributed along the grating- (*ε(z)*) as a function of the effective photoelastic constant (*P_e_*), thermo-optic (*ζ*) and thermal expansion (*α*) coefficients. In addition, *λ_0_* is the initial Bragg wavelength.

(6) $$\lambda_{B}=\lambda_{0}\left\{1+\left[\left(1-P_{e}\right)\varepsilon (z)+\left(\alpha +\zeta \right)\Delta T\right]\right\}$$

After knowing the forward and backward modes, the grating reflectivity (r) is estimated through Equation (7), which resulted in a differential equation to be solved using the transfer matrix approximation (see Equation (8)). Using this approximation, the grating length (L) is divided in M smaller sections and each section has uniform coupling properties. Table I shows the parameters used on the numerical analysis of the sensor’s operation principle.

(7) $$r(\lambda )={\left|\frac{S\left(\frac{-L}{2}\right)}{R\left(\frac{-L}{2}\right)}\right|}^{2}$$

(8) $$\left[\begin{array}{c} R_{i}\\ S_{i} \end{array}\right]=\left[\begin{array}{cc} \cosh\left(\gamma \Delta z\right)-i\frac{\sigma }{\gamma }\sinh\left(\gamma \Delta z\right) & -i\frac{k}{\gamma }\sinh\left(\gamma \Delta z\right)\\ i\frac{k}{\gamma }\sinh\left(\gamma \Delta z\right) & \cosh\left(\gamma \Delta z\right)+i\frac{\sigma }{\gamma }\sinh\left(\gamma \Delta z\right) \end{array}\right]\left[\begin{array}{c} R_{i-1}\\ S_{i-1} \end{array}\right]$$

For the numerical analysis, the spectra were divided in 200 sections. Furthermore, a constant strain of 500 με was applied on the half of the grating (L/2) in order to simulate the effect of its embedment in the 3D-printed structure, whereas no strain is applied on the other half of the FBG. Such strain value was obtained in a numerical simulation of the strain on the fiber when it is embedded in a TPU structure using the finite element method (such as the one presented in [12]). Figure 1 shows the simulated spectra for the half-embedded and unembedded cases, and a schematic representation of the sensor structure is also shown. The parameters used in the simulations are presented in Table 1.

The spectra presented in Figure 1 show the aforementioned redshift and reflectivity reduction caused by the embedment. Furthermore, there is a division of the grating spectrum in the half-embedded case, where one peak is related to the unembedded and the other to the embedded sections of the FBG. The FBG embedment leads to a variation on its force and temperature sensitivities (as depicted in [12]). For this reason, it is possible to assume that the embedded and unembedded peaks will have different sensitivities with respect to strain, transverse force and temperature. In order to theoretically evaluate the behavior of the FBGs under different parameters, we performed the additional simulations with the half embedded-FBG with the variation of strain, temperature and force. Due to the low Young’s modulus of the TPU (about 40 MPa), we consider that the TPU structure will not lead to a high reduction of the transverse force sensitivity of the embedded-FBG peak. Thus, for this simulation, we consider both peaks with same force sensitivity. On the other hand, the strain sensitivity depends on the TPU material properties, where the sensitivity of the embedded peak will be related to the strain distribution on the 3D-printed structure resulting in a higher sensitivity as presented in Equation (9) [7].

(9) $$K_{TPU}=K_{fiber}{\left(\frac{E_{TPU}}{E_{fiber}}\frac{d_{TPU}}{d_{fiber}}\right)}^{-1}$$

where K_fiber_ is the strain sensitivity of the FBG in silica optical fiber, K_TPU_ is the strain sensitivity of the FBG embedded in the TPU structure, E is the Young’s modulus (subscripts TPU and fiber indicate the Young’s modulus of the TPU and fiber, respectively). Similarly, d_TPU_ is the diameter of the TPU structure and d_fiber_ is the diameter of the optical fiber.

Similarly, the FBG embedded in a TPU structure also has higher temperature sensitivity when compared with the unembedded region of the FBG. The reason for this behavior is the high thermal expansion coefficient of the TPU, which leads to a thermally induced strain on the FBG when the temperature increases as demonstrated in [13]. Thus, the sensitivity for the embedded region of the FBG (K_TPU,T_) can be described with Equation (10) considering TPU thermal expansion coefficient (α_TPU_).

(10) $$K_{TPU,T}=\lambda_{0}\left[(\alpha +\zeta )+(1-P_{e})\alpha_{TPU}\right]$$

**Figure 2:**
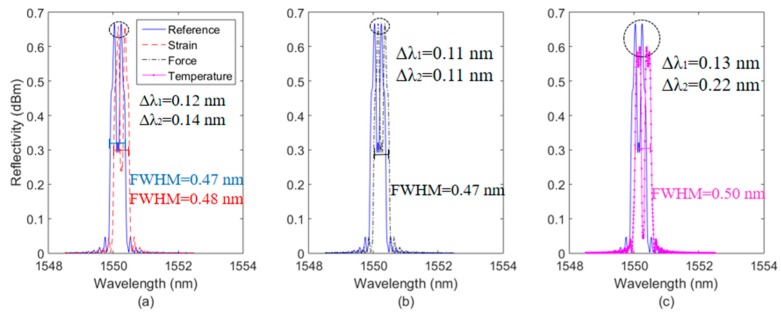
Simulated spectra of half-embedded FBGs for (**a**) strain variation of 100 µε, (**b**) force variation of N and (**c**) temperature variation of 15 °C.

Figure 2 shows the spectra simulations at different strain, temperature and transverse forces. In this case, the spectrum in the blue color is a reference spectrum, i.e., a spectrum without variation of strain, temperature and force, whereas the other spectra are the ones for strain (Figure 2a), force (Figure 2b) and temperature (Figure 2c) variations.

The results in Figure 2 indicate that there is a variation of the FWHM when the strain and temperature increase, since the embedded and unembedded regions of the FBG have different sensitivities as function of these parameters. For the transverse force variation, the FWHM does not change, since we consider that both peaks have the same sensitivity. It is also possible to observe the difference on the wavelength shift of each peak with respect to the applied parameters. Thus, if each peak and FWHM have different sensitivities (K_1_, K_2_ and K_FWHM_) with respect to different parameters (ε, F and T), it is possible to apply the direct difference technique (as shown in [7]) to obtain the response of each parameter considering the wavelength shift of each peak (Δλ_1_ and Δλ_2_) and FWHM as follows:

(11) $$\left[\begin{array}{c} \Delta \lambda_{1}\\ \Delta \lambda_{2}\\ \Delta FWHM \end{array}\right]=\left[\begin{array}{ccc} K_{\varepsilon ,1} & K_{F,1} & K_{T,1}\\ K_{\varepsilon ,2} & K_{F,2} & K_{T,2}\\ K_{\varepsilon ,FWHM} & K_{F,FWHM} & K_{T,FWHM} \end{array}\right]\left[\begin{array}{c} \Delta \varepsilon \\ \Delta F\\ \Delta T \end{array}\right]$$

## 3. Results and Discussion

**Figure 3:**
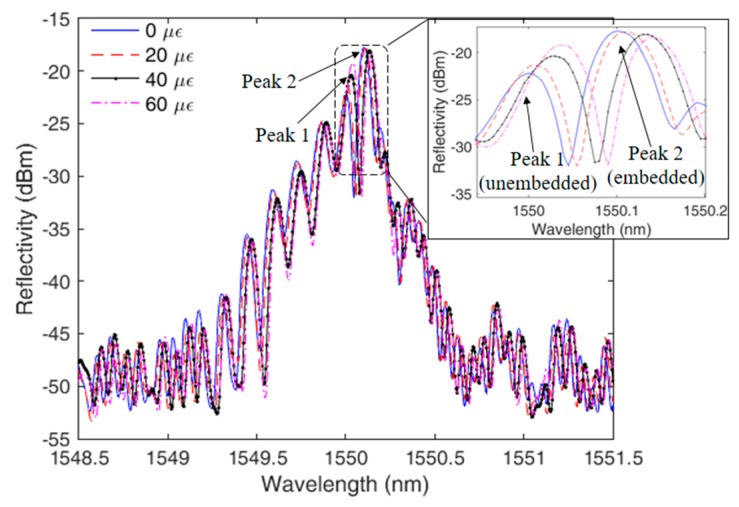
Half-embedded FBG spectra at different strains. Inset shows the magnified view of the two peaks created on the FBG embedment.

The sensor depicted in Section 2 is tested in different conditions of strain, temperature, and force in order to show the response of each peak as a function of those parameters. The TPU structure with half-embedded FBG is positioned on a linear translation stage and the spectral behavior is verified under low strain condition (strains up to 60 με). Figure 3 presents the FBG spectra at different strains, namely 20 με, 40 με and 60 με, where the figure inset shows a magnified view of the peaks generated at the half-embedding process. The peak 1 in the Figure 2 inset refers to the unembedded half of the grating, whereas the peak 2 is the one of the half embedded in the TPU structure. Compared with the simulations results (see Figure 1), the peaks presented a higher asymmetry due to non-uniform stress distribution on the grating during the embedment, which was not considered in the grating spectra simulation. In addition, the FBG reflectivity was reduced in about 40% after the embedment process, which is close to the reflectivity reduction predicted by the simulations.

**Figure 4:**
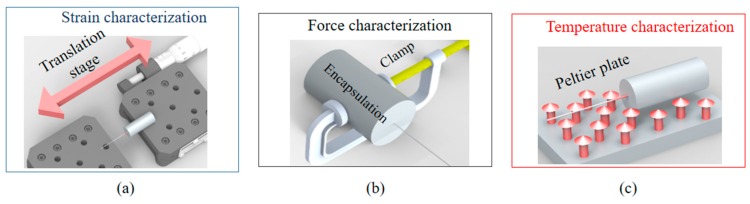
Experimental setup for the half-embedded TPU structure in the tests of (**a**) strain, (**b**) transverse force and (**c**) temperature.

The spectra shown in Figure 3 indicates that the shift on the FBG peaks occurs at different rates (sensitivities). In order to quantitatively verify such difference on the peaks responses, Figure 4 presents the experimental setup used on the sensor characterizations, where Figure 4a presents a schematic representation of the setup used on the strain tests at room temperature and without transverse force. Similarly, Figure 4b,c depict the experimental setup force (at room temperature, without applied strain) and temperature (without application of strain and transverse force) characterizations, respectively. 

**Figure 5:**
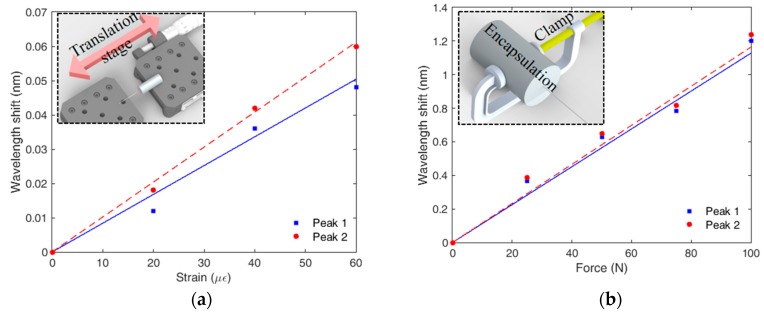

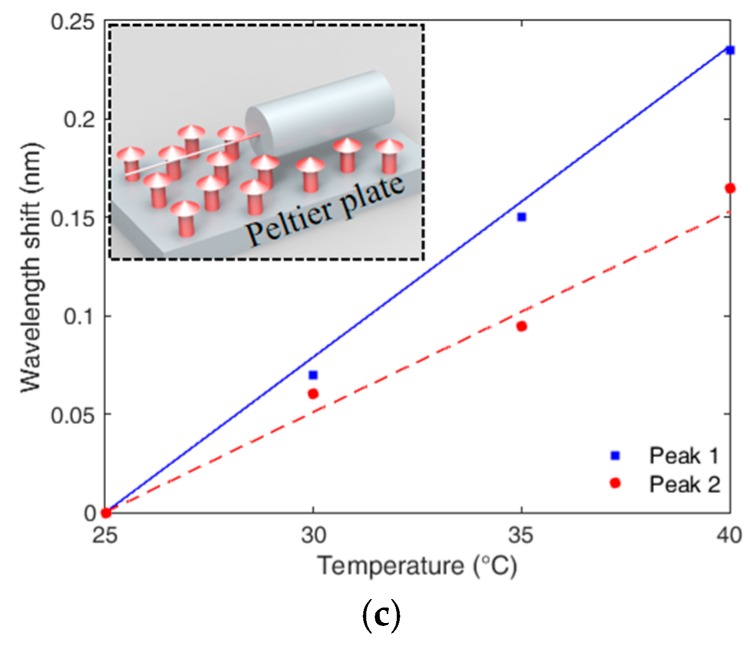
Wavelength shift for the half-embedded TPU structure in the tests of (**a**) strain, (**b**) transverse force and (**c**) temperature.

In Figure 5a, the lower sensitivity of the peak 1 (1.11 pm/με) noticeable when compared with the slope of the response of peak 2 (1.4 pm/με), which is due to the embedded material response as described in Equation (9). For the temperature tests presented in Figure 5c, the TPU structure with half-embedded FBG is positioned over a thermoelectric Peltier plate (TEC1-12706, Hebei IT) with a temperature controller (TED 200 C, Thorlabs). As discussed in [13], the FBG embedment in polymer structures (such as the TPU structure) leads to an increase of the sensor sensitivity due to the thermal expansion of the polymer structure. Thus, peak 2 has higher temperature sensitivity (15.7 pm/°C) than the unembedded half (peak 1), where the temperature sensitivity is 10.9 pm/°C. In contrast, the force sensitivity of both peaks is similar obtained on the results presented in Figure 5b, where the sensitivities are about 12.4 pm/N and 11.6 pm/N for peaks 1 and 2, respectively. The tests were made by encapsulating the TPU structure in another flexible structure in such a way that the applied force is transmitted to both embedded and unembedded half of the FBG, where the force is applied on the structure through the clamps depicted in Figure 5b. As discussed in [12], the FBG embedment reduces the force sensitivity but increases the dynamic range. Thus, the embedded peak has lower sensitivity than the unembedded one. However, it is worth to mention that the FBG broke on the unembedded section when a force higher than 100 N was applied on the sensor due to the brittle nature of silica fibers. All tests were performed at constant relative humidity conditions, where the relative humidity was about 70%.

**Figure 6:**
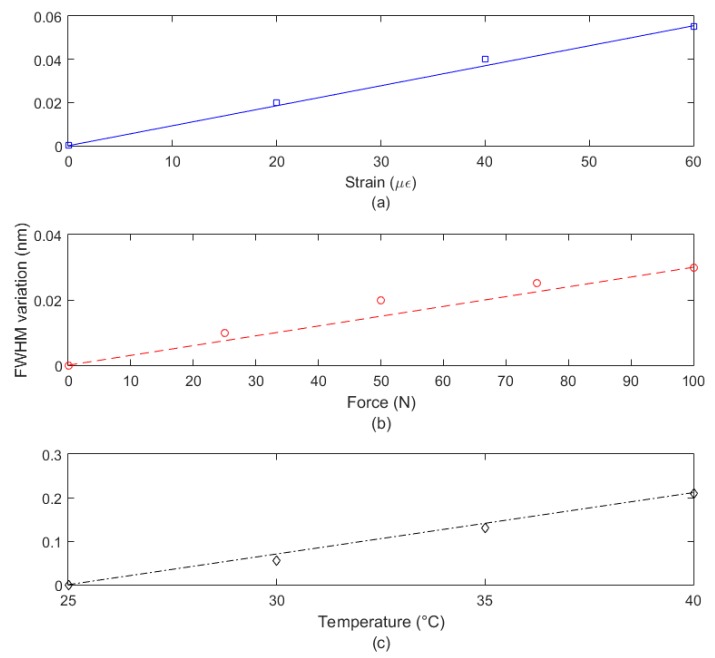
FWHM variation for the half-embedded TPU structure in the tests of (**a**) strain, (**b**) transverse force and (**c**) temperature.

In addition, Figure 6 shows the FWHM variation as a function of strain, force and temperature, where it is possible to observe that the FWHM has higher sensitivity to temperature variations due to the higher differences between the sensitivities of each peak in this case. Similarly, the lowest variation was found in the transverse force analysis due to the similar force sensitivity that both peaks presented. Since peak 2 is the one with higher sensitivity with respect to strain, force and temperature, there is an increase of the FWHM in all analyzed cases (due to the higher shift of peak 2 than peak 1). However, this shift is small for the force assessment (about 0.03 nm), where both peaks have similar sensitivities.

After characterizing the sensor sensitivities for each parameter, we analyze a multiparameter sensor for simultaneous measurement of temperature and strain and transverse force. Thus, the setups presented in Figure 4 are used, where the temperature is known using a temperature reference sensor positioned on the Peltier plate. It is worth noting that all tests were performed at the same humidity conditions of about 70% (room humidity). The analysis for simultaneous measurement of temperature, strain and force are performed in the same ranges as the ones employed on the sensors characterization tests (see Figure 5). In order to obtain the responses for temperature, strain and force, the sensor sensitivities (obtained from Figure 5 and Figure 6) are applied in Equation (11), where the equation is used for obtaining strain, force and temperature. In order to summarize the parameters used in Equation (11) for the multiparameter measurement, Table 2 presents the sensor sensitivities with respect to each analyzed parameter.

**Figure 7:**
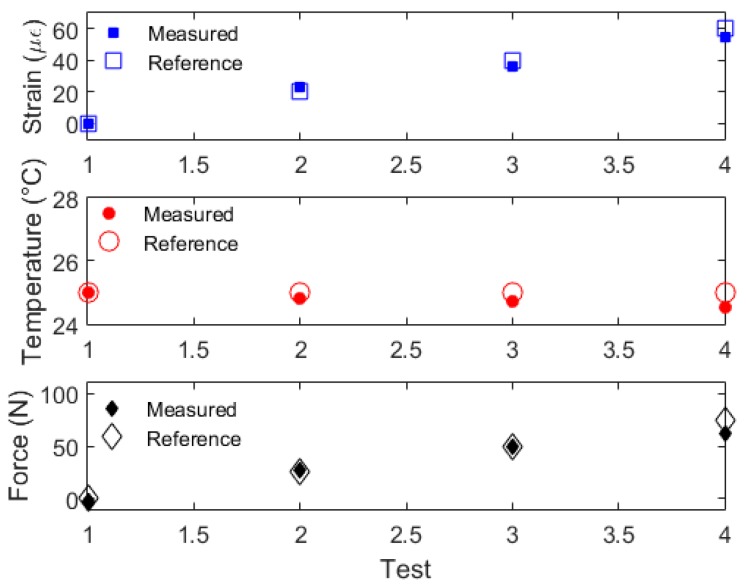
Strain, temperature and force estimations with the proposed FBG embedded in a TPU structure.

The sensitivities depicted in Table 2 are applied in Equation (11) in order to obtain the sensors responses as function of each parameter. Figure 7 shows the sensor responses for multiparameter sensing. Moreover, the reference strain, force and temperature are also presented in Figure 7 for comparison purposes, where such references were obtained from the translational stage, clamp and room temperature sensors, respectively.

Regarding to the results presented in Figure 7, it is possible to observe the feasibility of the proposed sensor system on the assessment of multiple environmental and mechanical parameters. Comparing with the reference, the proposed sensor system shows a root mean squared error (RMSE) of 3.50 με, 0.27 °C and 6.11 N for strain, temperature and force, respectively. The presented RMSEs result in a mean relative error of 4%, considering the mean errors of all parameters. As the determinant of the K-matrix (see Equation (11)) is not zero as can be inferred from the coefficients presented in Table 2, the proposed technique is applicable and can measure all three parameters (strain, temperature and force) simultaneously, since the Equation (11) will result in a linear equation system. Such system has 3 equations (one for each parameter), 3 unknowns (temperature, strain and force) and 3 measured variables (wavelength shift of peak 1 and peak 2 as well as the FWHM), which, from linear algebra, results in a determined system, i.e., a system with only one group of solutions, leading to a defined temperature, strain and force estimation for each spectral condition. However, if the combination of peak 1, peak 2 and FWHM leads to multiple temperature, force and strain solutions, it is possible to eliminate this multiplicity of solutions by analyzing the previous signals (or signals derivative) and comparing it with the dynamics of the system at which the proposed sensor system will be applied. In this case, solutions that result in parameter variations with a rate higher than the ones that the system is able to provide (due to its dynamics) will not be considered. 

## 4. Conclusions

In this paper, we presented the numerical analysis and experimental validation of a half-embedded FBG. Half of the grating length was embedded in a 3D-printed TPU structure, which resulted in a division of the FBG spectrum. In this case, two peaks are created and the experimental results of the proposed sensor as function of temperature, strain and force have shown different sensitivities of the peaks as well as the variation of the FWHM as function of these different parameters. Therefore, it is possible to perform simultaneous measurements of different parameters using only one FBG by applying Equation (11) on the response of both peaks by combining all of the three parameters tested, the determinant of the matrix shown in Equation (11) is not null, which is the condition for applying such equation for simultaneous measurement (as discussed in [19]). The results for the simultaneous measurement show the feasibility of the proposed sensor system in measuring all three parameters simultaneously with relative errors as low as 4%. In future investigations, we envisage the use of this technique in simultaneous measurements of different parameters in robotics applications.

## Figures and Tables

**Table 1:** Parameters employed in the gratings simulation.

Parameter	Symbol	Value
Nominal period	Λ*_0_*	536 nm
Fringe visibility	*ν*	1
Initial wavelength	*λ_0_*	1550 nm
Photoelastic constant	*P_e_*	0.22
Effective refractive index	*n_eff_*	1.4455
Number of grating sections	*M*	200

**Table 2:** Sensitivities of peaks 1 and 2 for temperature, strain and force.

Parameter	Symbol	Value
Temperature sensitivity, peak 1	*K_T,1_*	10.6 pm/°C
Temperature sensitivity, peak 2	*K_T,2_*	15.7 pm/°C
Strain sensitivity, peak 1	*K_ε,1_*	1.11 pm/με
Strain sensitivity, peak 2	*K_ε,2_*	1.40 pm/με
Force sensitivity, peak 1	*K_F,1_*	12.4 pm/N
Force sensitivity, peak 2	*K_F,2_*	11.6 pm/N
Strain sensitivity, FWHM	*K_ε,FWHM_*	1.1 pm/ με
Force sensitivity, FWHM	*K_F,FWHM_*	−0.3 pm/N
Temperature sensitivity, FWHM	*K_T,FWHM_*	14.1 pm/°C
